# Surface excitations in the modelling of electron transport for electron-beam-induced deposition experiments

**DOI:** 10.3762/bjnano.6.129

**Published:** 2015-06-03

**Authors:** Francesc Salvat-Pujol, Roser Valentí, Wolfgang S Werner

**Affiliations:** 1 Institut für Theoretische Physik, Goethe-Universität Frankfurt, Max-von-Laue-Straße 1, 60438 Frankfurt am Main, Germany; 2 Institut für Angewandte Physik, Technische Universität Wien, Wiedner Hauptstraße 8-10/134, 1040 Wien, Austria

**Keywords:** focused-electron-beam-induced deposition (FEBID), Monte Carlo simulation of electron transport, surface excitations, secondary-electron emission

## Abstract

The aim of the present overview article is to raise awareness of an essential aspect that is usually not accounted for in the modelling of electron transport for focused-electron-beam-induced deposition (FEBID) of nanostructures: Surface excitations are on the one hand responsible for a sizeable fraction of the intensity in reflection-electron-energy-loss spectra for primary electron energies of up to a few kiloelectronvolts and, on the other hand, they play a key role in the emission of secondary electrons from solids, regardless of the primary energy. In this overview work we present a general perspective of recent works on the subject of surface excitations and on low-energy electron transport, highlighting the most relevant aspects for the modelling of electron transport in FEBID simulations.

## Introduction

An accurate modelling of the energy losses of electrons traversing a solid surface is instrumental for a quantitative understanding of a series of techniques exploiting transmitted, reflected, or emitted electrons, including a number of spectroscopies (electron-energy-loss spectroscopy, X-ray photoelectron spectroscopy, and Auger-electron spectroscopy), electron microscopy, and the focused-electron-beam-induced deposition (FEBID) of nanostructures, on which we focus here. This technique employs beams of focussed kiloelectronvolts-electrons to trigger and steer the growth of nanostructures with tunable electronic and magnetic properties from molecules of organometallic precursor gases [1] adsorbed on a substrate [2]. It has been shown that, for irradiation with electrons of 1–5 keV, both the incoming primary electrons and the emitted secondary electrons influence the growth of the nanostructures, the latter electrons being responsible for the lateral resolution [3].

In the modelling of electron transport for FEBID [2–7], electron stopping is described on the basis of properties that are applicable in the bulk of the material. However, electrons traversing a solid interface additionally excite surface modes, an energy-loss channel that amounts to a sizeable fraction of the energy-loss spectrum for electrons of up to a few kiloelectronvolts. The existence of surface excitations was predicted in the late 1950s [8]; experimental evidence was obtained shortly thereafter [9–10]. In order to model surface excitations in electron spectroscopies, several models have been developed to date [11–31]. Various approaches are considered, often with underlying simplifications, evoked on physical or technical grounds, in the interest of making calculations feasible in a finite time. In order to derive a distribution of energy losses of charged projectiles moving in the vicinity of the surface, some of the models cited above rely on the semiclassical dielectric formalism, whereas others adopt a many-body formalism. Both approaches have been shown to yield results in equivalently good agreement [32] with experimental data.

In what follows we briefly review the stopping of charged projectiles in the vicinity of a solid surface, along the lines of [31], which will be referenced for further details. We summarize a series of rules which characterize the behavior of the probability for surface excitations and we briefly review a practical model for the emission of secondary electrons. Relevant aspects to FEBID modelling will be highlighted.

## Review

### Inelastic collisions in the bulk of the material

Energy losses of a charged projectile moving in a solid can be described accurately within the semiclassical dielectric formalism. In this approach, one assumes that the presence of the charged projectile disturbs the equilibrium charge density of the solid, which becomes polarized and, thus, an electric field is induced at all points of space. The force acting on the charged projectile due to the induced electric field is assumed to be the agent responsible for its (electronic) stopping. In order to derive physical quantities that describe the stopping, it is now a matter of calculating first the induced electric field and, from it, the so-called stopping power, defined as the variation of the kinetic energy of the projectile per unit path length. Once an expression for the stopping power is derived, one can identify from it an expression for the distribution of energy losses per unit path length, the basic quantity that is needed to describe energy losses in a detailed Monte-Carlo simulation of electron transport. In this section we briefly outline the basic steps of these calculations and highlight the underlying assumptions. Further details can be found in the cited works.

The starting point of the calculation is the dielectric function ε(*q*,ω) of the material, where *q* and ω are the respective Fourier-conjugate variables of the position, **r**, and the time, *t*. In practice one typically has data available for ε(ω), be it from optical data obtained experimentally [33] or from theoretical calculations, e.g., via density-functional theory calculations [33–35]. An ω-dependent dielectric function is sufficient to describe the response of the medium to a spatially homogeneous perturbation, such as that of an incoming photon. However, for incoming charged projectiles the perturbation is strongly dependent on the spatial coordinates, so that a *q*-dependent dielectric functions is required. Physically reliable models are built on the basis of the (*q*,ω)-dependent dielectric function for the homogeneous electron gas [36–38] or on the basis of a simple superposition of Drude–Lindhard oscillators [33].

Assuming a projectile that moves with a velocity **v** along a trajectory **r** = **v***t*, one can conveniently solve the Maxwell equations in Fourier space to obtain the following expression for the induced electric field [31]

[1]
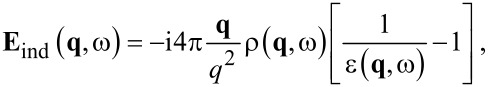


where ρ(**q**,ω) is the Fourier transform of the projectile charge density ρ(**r**,*t*) = *Z*_0_*e*δ(**r**−**v***t*), where *Z*_0_ is the projectile charge in units of the modulus of the electron charge, *e*, and **v** is the velocity of the projectile. To obtain this expression, the following approximations were considered: (1) The Coulomb gauge was adopted and the contribution from the vector potential was neglected. (2) The dielectric displacement field was assumed to be proportional to the electric field in Fourier space (linear response). The first approximation restricts the validity of the calculation to non-relativistic projectiles (the calculation with the full electric field for relativistic projectiles is also feasible [39]), whereas the second approximation can be seen to be formally equivalent to a first-order Born approximation in perturbation theory, imposing a lower bound to the domain of validity of the calculation [22,40], which for practical purposes is above 100 eV.

The stopping power *S* is obtained as the variation of the kinetic energy per unit path length,

[2]
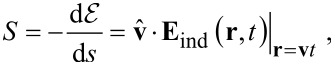


where 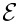 is the kinetic energy of the projectile and *s* = *vt* is the path length. Combining Equation 1 and Equation 2 one obtains

[3]
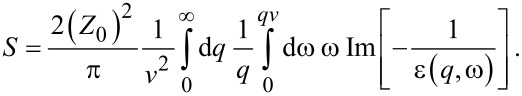


Up to this point the stopping of the projectile is treated as a continuous phenomenon, whereas in reality charged projectiles lose energy and are deflected in the course of individual inelastic collisions. The so-called semiclassical approximation consists in assigning to 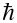
**v** and 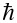
ω the meaning of a momentum transfer from the projectile to the medium and of an energy loss of the projectile, respectively. Atomic units (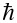 = *m_e_* = *e* = 1) will be used below. Now that these variables have a well-defined physical meaning, the corresponding integrals must be restricted to the kinematically allowed domain,

[4]
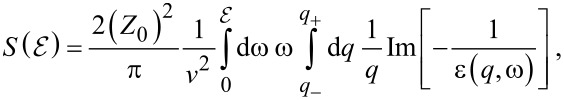


where

[5]
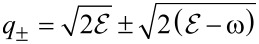


are the minimum (−) and maximum (+) momentum transfers allowed by the energy and momentum conservation laws.

Equation 4 can be understood as the average energy loss per unit path length dictated by a distribution of energy losses per unit path length, dμ/dω:

[6]
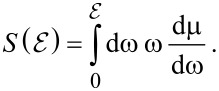


The quantity dμ/dω is known as the differential inelastic inverse mean free path (DIIMFP), explicitly given by

[7]
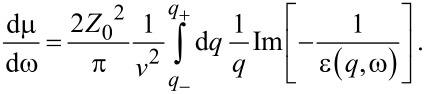


Note that the DIIMFP is a function of the energy loss for the given velocity of the projectile. The integral of the DIIMFP over all allowed energy losses gives the inelastic inverse mean free path

[8]
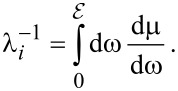


The latter two quantities are the necessary quantities for a detailed Monte-Carlo simulation of electron transport (see section “Monte-Carlo simulation of electron energy-loss spectra”), a method that has been successfully used in the last decades.

### Inelastic collisions in the vicinity of a planar surface

The scheme outlined in the previous section to describe inelastic interactions of charged projectiles in solids gives a good account of inelastic collisions in the bulk of the solid. However, projectiles impinging and emerging from a solid additionally cross a planar interface to vacuum (or another solid) that is not explicitly accounted for. The existence of a planar surface imposes additional boundary conditions on the electric field [41–42].

Several approaches exist in the literature to solve the Maxwell equations with these boundary conditions for the stopping problem: Some consider the dielectric function of a semi-infinite medium [43], and others (preferred in the electron-spectroscopy community) rely on a method that allows one to work with bulk dielectric functions, the method of image charges, also known as the method of extended pseudomedia. The method consists in rephrasing the semi-infinite-geometry problem as the sum of two infinite-geometry problems, supplied with a series of fictitious charges that are determined in terms of known quantities by imposing the boundary conditions at the interface.

The resulting induced electric field has a more complex expression than in the bulk case. Nevertheless, it can be expressed as the sum of one contribution arising from a charge density induced in the bulk of the material and another one arising from a charge density induced at the surface of the material.

The DIIMFP resulting from the induced electric field becomes more complicated, with two additional parametric dependencies: (1) on the depth coordinate with respect to the surface and (2) on the surface crossing angle with respect to the surface normal. Several models exist with varying approximations [23–24,30–31], the effects of which were scrutinized [31]. Regardless of the details of the models, they all yield a number of consistent general features and trends of the surface excitation probability:

- Surface energy losses can be undergone by the charged projectile on either side of the interface, at the solid side or at the vacuum side. Indeed, a surface charge can be induced regardless of the side at which the projectile is moving on and, thus, a charged projectile moving on the vacuum side of the interface can also undergo energy losses. It has been recently shown that, in reflection-electron-energy-loss spectra, surface losses on the vacuum side of the interface account for a large fraction of the surface-excitation intensity, often more than half of it [44].
- The probability for an electron that crosses a surface to undergo a surface excitation is, to a first approximation [45], proportional to the surface dwell time 
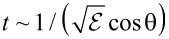
, where 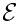 is the projectile energy and θ is the surface crossing angle with respect to the surface normal. The energy dependency implies that, in practice, surface excitations are relevant for electron energies up to a few kiloeelectronvolts. Additional structure to the aforementioned angular behavior is predicted for scattering geometries coinciding with deep minima of the differential elastic scattering cross section: minor deflections in the course of an inelastic collision lead to an effective scattering geometry with enhanced elastic scattering and therefore higher detection probability [44].
- The DIIMFP for energy losses of charged projectiles impinging on a surface differs from the DIIMFP for the conjugate emerging direction. This effect, known as in-out asymmetry in surface energy-losses, has been long predicted but only recently observed experimentally [46]. In-out differences are most accentuated for surface-crossing directions close to the surface normal and for high kinetic energies (about 1 keV).

### Monte-Carlo simulation of electron energy-loss spectra

The electron-transport problem in a solid is described in terms of a Boltzmann-type transport equation. A practical method for solving the problem is provided by Monte-Carlo simulation, which consists in sampling an ensemble of trajectories undergoing collisions of the relevant types as dictated by a given set of interaction cross sections. A statistical average of the desired observable is performed over the sampled trajectories to the selected precision [47].

**Figure 1:**
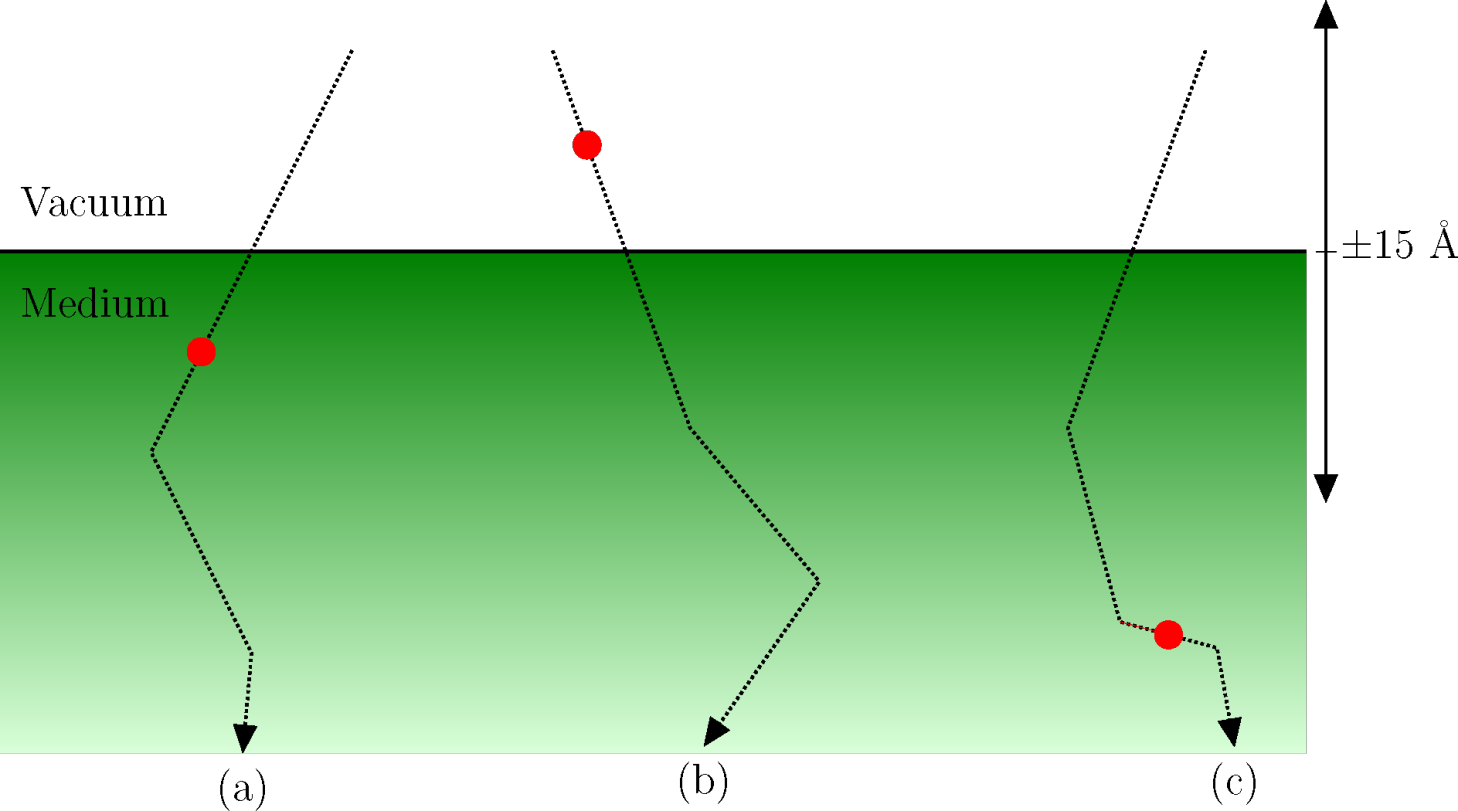
Example incoming trajectories (dotted lines) in the surface-scattering zone (typically 15 Å above and below the surface), undergoing (a) a surface energy loss in the medium side, (b) a surface energy loss in the vacuum side, (c) a bulk energy loss, indicated by the filled circles.

**Figure 2:**
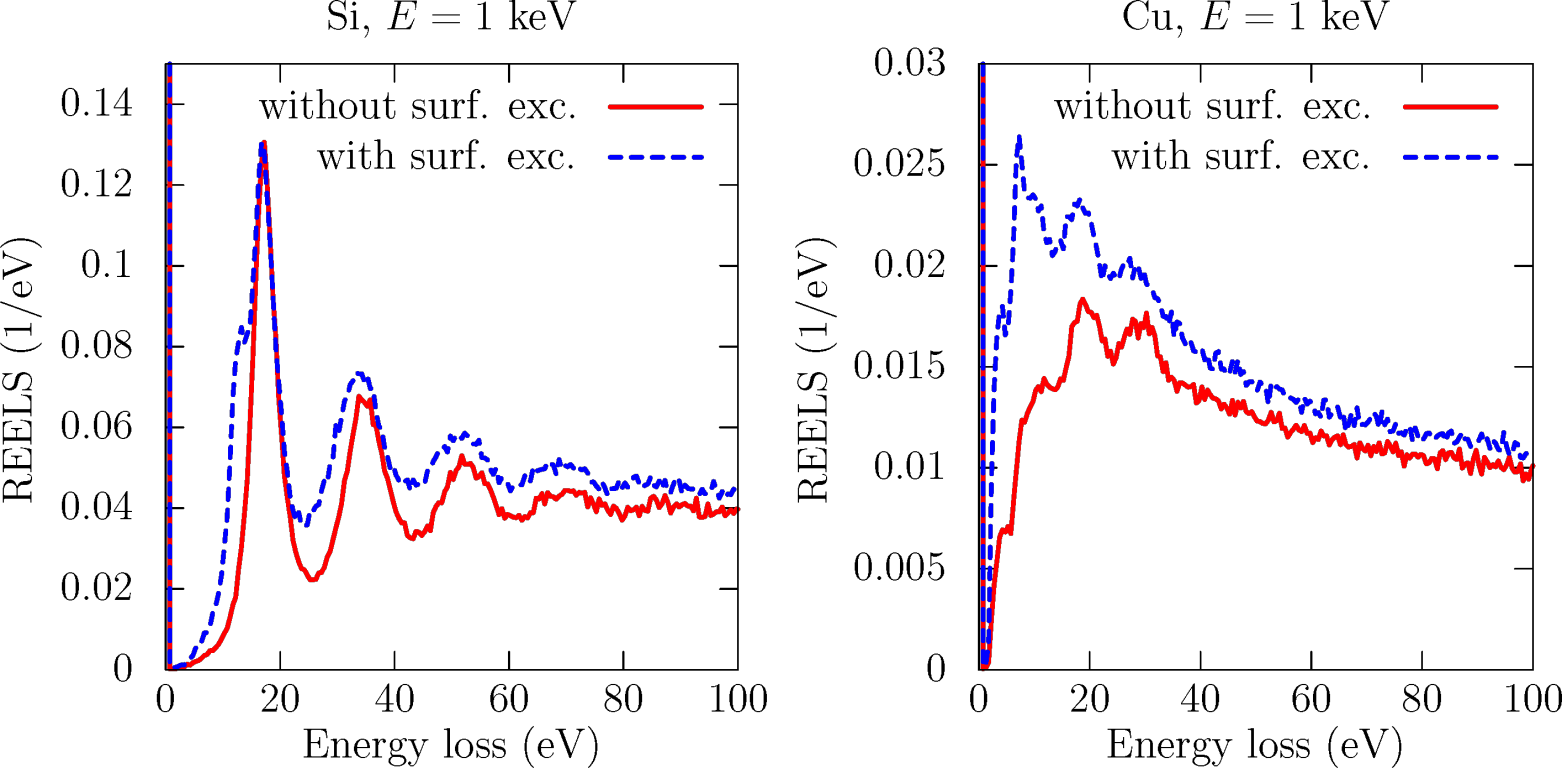
Comparison of reflection-electron-energy-loss spectra (REELS) of Si (left) and Cu (right) under bombardment with 1-keV electrons at normal incidence, without (red solid curves) and with (blue dashed curves) an account of surface excitations. All backscattered electrons are collected.

**Figure 3:**
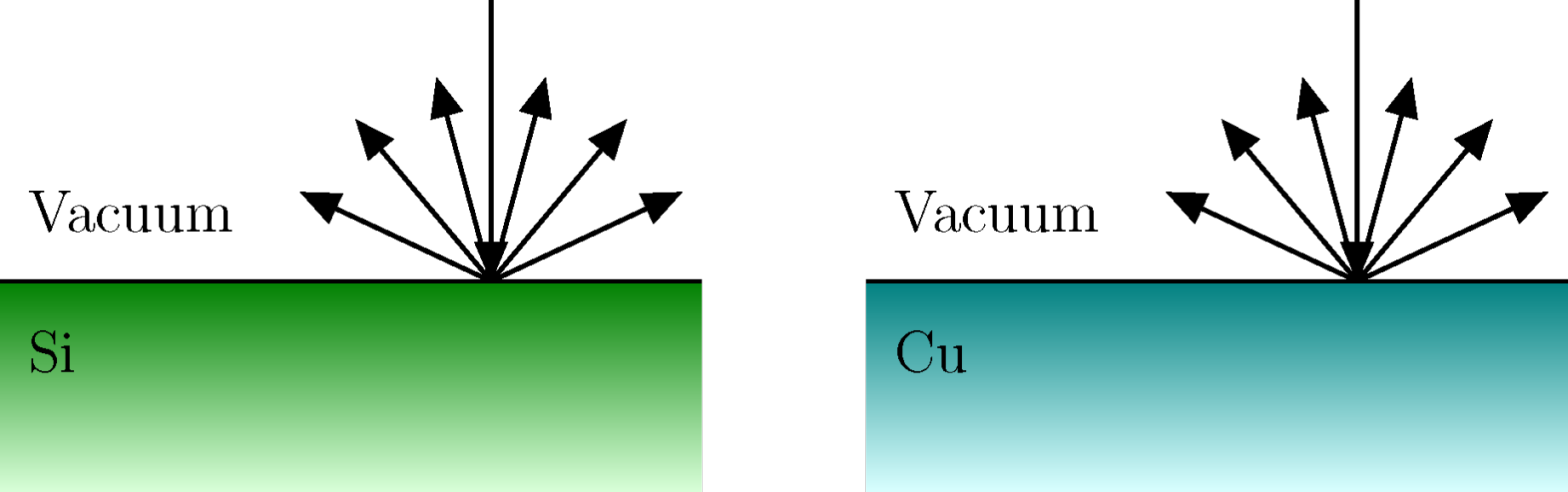
Simulation geometry: 1 keV electrons impinge normally onto the material (Si or Cu); all backscattered electrons are collected.

In the energy range between 100 eV and a few keV the relevant interaction mechanisms of electrons with the solid are elastic collisions with the atoms and inelastic collisions with typically weakly bound electrons in the solid. Elastic scattering can be accurately described by means of a differential cross section for elastic scattering (DCES), which can be systematically calculated by means of partial-wave calculations [48–49]. Inelastic scattering is accounted for by the DIIMFPs described above. Monte-Carlo simulations of electron transport (bulk losses only) for typical geometries in FEBID experiments have been previously considered [2,7]. The inclusion of surface excitations implies a modification of the sampling algorithm in the vicinity of the surface (typically 15 Å above and below the surface), as schematically shown in Figure 1. Technical details on the implementation of the algorithm for the simulation of surface energy losses can be found elsewhere in great detail [30–31,50]. Here the focus is on the effect of surface excitations on the reflection-electron-energy-loss spectrum (REELS). To this effect, Figure 2 compares the REELS of Si (left) and Cu (right) under bombardment with 1 keV electrons impinging perpendicularly on the sample; all reflected electrons are collected. The simulation geometry is depicted in Figure 3. The materials are chosen as representative substrate (Si) and deposit (Cu) materials. The solid red curves (dashed blue curves) in Figure 2 correspond to REELS simulated without (with) the inclusion of surface excitations. We observe that even for a primary energy of 1 keV surface excitations account for (1) additional features, i.e. the excitation of surface plasmons, in the low-energy-loss part of the REELS that are not accounted for by a bulk-only description of the energy losses of charged projectiles in the material and (2) a sizeable fraction of the intensity in the first few tens of eV of energy losses, about 20% of the intensity in the case of Si and 15% of the intensity in the case of Cu. Although the relative importance of surface excitations is enhanced for lower energies, their effect is noticeable even in the 1 keV domain. Thus, the inclusion of surface excitations in the modelling of electron-transport is expected to give a yet more quantitative description of FEBID processes at and below the 1 keV primary-energy domain.

### Secondary-electron emission

**Figure 4:**
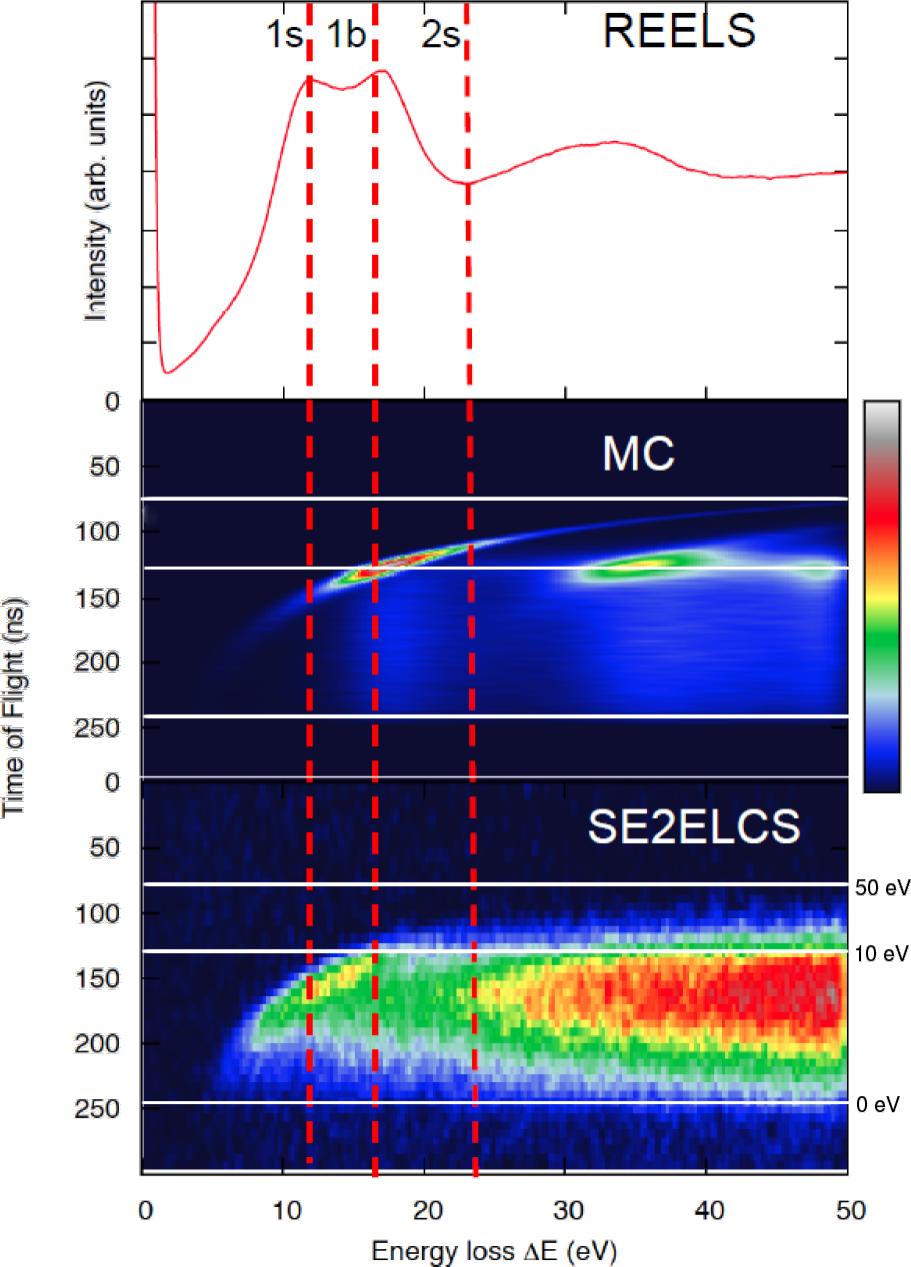
(Upper panel) Reflection-electron-energy-loss spectrum (REELS) of Si under 100 eV bombardment (see [55] for experimental details). (Lower panel) (e,2e)-coincidence spectrum of secondary electrons emitted in coincidence with energy losses (SE2ELCS) of 100-eV electrons backscattered from Si. (Middle panel) Monte-Carlo simulation of the SE2ELCS measurement without accounting for surface energy losses. The vertical dashed lines in red indicate energy losses corresponding to the excitation of one surface, one bulk, and two surface plasmons. The horizontal solid white lines indicate the times of flight corresponding to electrons with 0 eV (accelerating grids were used), 10 eV, and 50 eV.

Energy losses of the charged projectile can lead to the ejection of loosely bound electrons of the solid, which emerge as secondary electrons (SE). The majority of these SE are of relatively low energies (≤50 eV). These energies are well below the domain of validity of the elastic and inelastic interaction cross sections available in the literature, which has been a limitation for progress in the field. Electron coincidence measurements [51–54] have supplied a wealth of valuable information. Recently, coincidence measurements of correlated electron pairs emitted from solids (Al, Si, Ag) under electron bombardment have been measured, providing a double-differential SE yield, differential with respect to the energy loss of the primary electron and with respect to the energy (or the time of flight) of the emitted secondary electron [55]. These experimental data are displayed for Si under 100 eV electron bombardment in the lower panel of Figure 4 as a bird’s-eye-view plot (only the shape and relative intensities of the spectrum are of relevance here, hence the missing units in the linear color scale, where black is the null point and white is the maximum attained value). The horizontal white lines indicate the corresponding times of flight for electrons with 0 eV (accelerating grids were used), 50 eV, and 100 eV. See [55] for the experimental details. The plot can be read as the (time-of-flight) spectrum of secondary electrons emitted as a result of different energy losses of the impinging electron (to be read at the abscissae). The upper panel of Figure 4 displays the REELS of 100 eV from Si, where the energy-loss peaks corresponding to the excitation of one surface plasmon, one bulk plasmon, and two surface plasmons are indicated by vertical red dashed lines and labeled, respectively, 1s, 1b, 2s as a guideline for the abscissae scale in the other plots of the figure.

**Figure 5:**
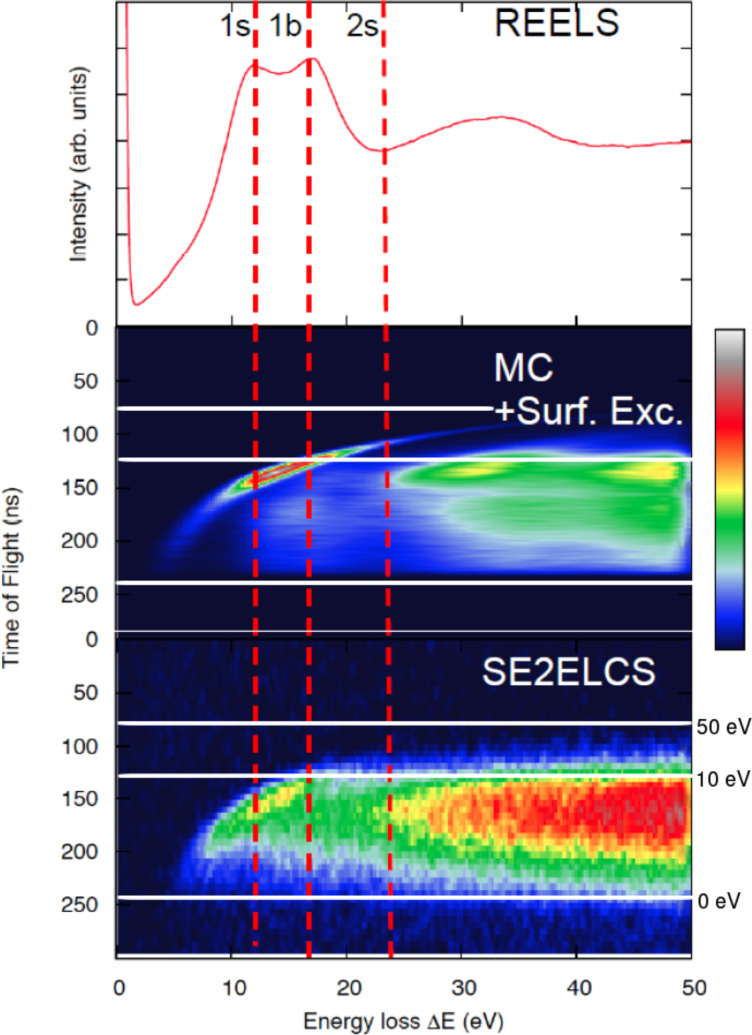
Same as Figure 4 with the inclusion of surface excitations in the modelling of electron transport through the solid-vacuum interface.

The coincidence data (e.g., lower panel of Figure 4) provide on the one hand very detailed insight into the mechanisms responsible for SE emission and, on the other hand, provide a benchmark against which models for SE emission and low-energy electron transport in general can be tested. The transport models, in turn, aid in the interpretation of the data, as discussed below. The Monte-Carlo simulation briefly outlined above was extended to include the generation and the transport of the secondary electrons and to simulate the electron-coincidence measurement on the basis of a simple model for SE emission: every time that the primary electron undergoes an energy loss, a SE trajectory is started with the energy loss as an initial energy (see [55] for the simulation details). Having the experimental data as a guideline, the interaction cross sections described above were used down to 1 eV (knowing that this is well below the domain where they are formally applicable) as a first approximation. Simulations were first carried out using bulk energy-loss DIIMFPs exclusively. The resulting spectrum is displayed in the middle panel of Figure 4. It is clear that these simulated peaks do not reproduce the onset of the experimentally observed peaks. Only after the inclusion of surface excitations, both for the incoming primary electrons, for the backscattered electrons, and for the emitted secondary electrons, is good agreement between simulations and measurements found, as shown in Figure 5. The Monte Carlo simulations further allow one to discern the processes that give rise to the different regions of the coincidence spectrum [55].

Thus, it was found that any realistic model of SE emission and low-energy electron transport near solid surfaces must account for surface excitations. This conclusion has strong implications on the emission depth from which SE are emitted: if secondary electrons undergo additional energy losses on their way out of the solid, the average SE-emission depth becomes much shallower than one would assume on the basis of a model based only on bulk properties. The predicted number of emitted SE can also differ appreciably with respect to a bulk-only simulation. Furthermore, the energies of the SE are also modified by the presence of additional surface energy-loss channels.

## Conclusion

In light of the presented richness in the behavior of surface excitations and their effect on both the energy losses of the impinging electrons and on the emission of secondary electrons, it is to be expected that their inclusion in the modelling of electron transport for FEBID will yield a more detailed description of the role played by both the primary electrons and the emitted secondary electrons in the growth process. It should be noted that, while surface excitations are relevant for primary electrons with energies up to 1–2 keV, they are essential ingredients for the modelling of slow secondary electrons regardless of the energy of the primary electron responsible for their emission.

The previous considerations suggest that the inclusion of surface excitations in the electron-transport model employed to investigate FEBID experiments might lead to noticeable effects. On the one hand, more primary electrons are backscattered compared to the case without surface excitations (see Figure 2), so that an increase in the simulated deposition rate might be expected (at least for primary energies in the 1–2 keV regime and below). On the other hand, more slow (≤50 eV) secondary electrons will be available from the decay of surface plasmons [56] excited by either the incoming electrons or the outgoing electrons (backscattered electrons or emitted secondary electrons). This should also contribute to an increase of the simulated deposition rate and additionally lead to an enhancement of the FEBID proximity effect.
